# Randomized controlled trial evaluating a virtual parenting intervention for young children at risk of obesity: study protocol for Parenting Addressing Early Years Intervention with Coaching Visits in Toronto (PARENT) trial

**DOI:** 10.1186/s13063-022-06947-w

**Published:** 2023-01-04

**Authors:** Sarah Rae, Jonathon Maguire, Mary Aglipay, Melanie Barwick, Karoon Danavan, Jess Haines, Jennifer Jenkins, Marie Klaassen, Myla E. Moretti, Frank Ong, Nav Persaud, Michelle Porepa, Sharon Straus, Erika Tavares, Andrew Willan, Catherine Birken

**Affiliations:** 1 Department of Nutritional Sciences, Faculty of Medicine, University of Toronto, Toronto, ON Canada; 2 Child Health Evaluative Sciences, The Peter Gilgan Centre for Research and Learning, The Hospital for Sick Children, Toronto, ON Canada; 3 Li Ka Shing Knowledge Institute, St Michael’s Hospital, Toronto, ON Canada; 4 Department of Family Relations and Applied Nutrition, University of Guelph, Guelph, ON Canada; 5 Dalla Lana School of Public Health, University of Toronto, Toronto, ON Canada; 6 Department of Psychiatry, University of Toronto, Toronto, ON Canada; 7 Department of Pediatrics, Department of Medicine, University of Toronto, Toronto, ON Canada; 8 Applied Psychology and Human Development, University of Toronto, Toronto, ON Canada; 9 Toronto Public Health, Toronto, ON Canada; 10 Institute of Health Policy Management and Evaluation, University of Toronto, Toronto, ON Canada; 11 Clinical Trials Unit, Ontario Child Health Support Unit, The Hospital for Sick Children, Toronto, ON Canada; 12 Department of Family and Community Medicine, Centre for Urban Health Solutions and Department of Family and Community Medicine, University of Toronto, St. Michael’s Hospital, Toronto, ON Canada; 13 Department of Pediatrics, St Michael’s Hospital, Pediatric Research, Toronto, ON Canada; 14 Genetics and Genome Biology, The Hospital for Sick Children, Toronto, ON Canada

**Keywords:** Randomized control trial, Intervention, Childhood, Obesity, Cohort, Evaluation

## Abstract

**Background:**

The prevalence of overweight (15%) and obesity (6%) in children under 5 years of age in Canada are high, and young children with overweight and obesity are at increased risk of the development of chronic disease(s) in adulthood. Prior research has demonstrated very few published trials on effective obesity prevention interventions in young children at risk of obesity, within primary healthcare settings. The aim of this study is to determine if 18–48-month-old children at risk for obesity, who are randomized to receive the Parents Together program (i.e., intervention group), have reduced body mass index *z*-score (zBMI), compared to those not receiving the intervention, at a 12-month follow-up. Secondary clinical outcomes between the intervention and control groups will be compared at 12 months.

**Methods:**

A pragmatic, parallel group, 1:1, superiority, randomized control trial (RCT) through the TARGetKids! Practice Based Research Network will be conducted. Young children (ages 18–48 months) who are at increased risk for childhood obesity will be invited to participate. Parents who are enrolled in the intervention group will participate in eight weekly group sessions and 4–5 coaching visits, facilitated by a trained public health nurse. Children and parents who are enrolled in the control group will receive the usual health care. The primary outcome will be compared between intervention arms using an analysis of covariance (ANCOVA). Feasibility and acceptability will be assessed by parent focus groups and interviews, and fidelity to the intervention will be measured using nurse-completed checklists. A cost-effectiveness analysis (CEA) will be conducted.

**Discussion:**

This study will aim to reflect the social, cultural, and geographic diversity of children in primary care in Toronto, Ontario, represented by an innovative collaboration among applied child health researchers, community health researchers, and primary care providers (i.e., pediatricians and family physicians in three different models of primary care). Clinical and implementation outcomes will be used to inform future research to test this intervention in a larger number, and diverse practices across diverse geographic settings in Ontario.

**Trial registration:**

ClinicalTrials.gov NCT03219697. Registered on June 27, 2017.

## Background

The prevalence of overweight (15%) and obesity (6%) [1, 2] in Canadian children are high, and one-third of Canadian youth are affected. Preschool children with overweight or obesity are at risk of obesity in later childhood and adulthood, mental health problems, cardiometabolic disease [3–5], and increased healthcare utilization [6].

Most obesity prevention interventions have been delivered in elementary or middle school settings [7]. Few interventions have been delivered in other settings such as home, primary care, or community settings. Home visiting, parenting skill-building, and parent support through coaching calls are all promising approaches toward obesity prevention in early childhood [8–13]. Two trials implemented intensive and frequent home visits and parenting support through coaching calls and text messages, showing a small but significant reduction in standardized zBMI at 6 months and 2 years [10, 11]. One RCT conducted in primary care used a group-based parenting intervention alone and demonstrated improvement in child’s eating behaviors but not zBMI [9]. Another trial where primary care providers delivered biweekly counseling and telephone phone coaching for parents found a reduction in total energy intake at 12 months among those in the intervention, but there was no difference seen in the children’s zBMI [12].

Many interventions that have demonstrated effectiveness were of high intensity and duration and utilized core components such as home visiting or telephone support in primary care, but few focused on children at risk of obesity. The PARENT trial aims to determine if a multifaceted intervention (including coaching visits and virtual group parenting sessions), delivered by a trained public health nurse, with a focus on parenting skills to support healthy behaviors, will result in reduced zBMI and improved health behaviors and outcomes when compared to usual care at 12 months post-intervention. Evaluation of implementation outcomes will inform clinical outcomes and the potential for future scale-up.

## Methods/design

### Participants and study design

#### Study design

This will be a pragmatic, parallel group, 1:1, superiority, randomized controlled trial, within The Applied Research Group for Kids (TARGetKids!) Practice Based Research Network. TARGetKids! is a primary care research network for children in Toronto, Montreal, and Kingston, Canada. The protocol for this study has been developed in accordance with the *SPIRIT Reporting Guidelines* [13].

#### Inclusion criteria

Young children (18–48 months) identified as being at increased risk for childhood obesity will be eligible and invited to participate. The following risk factors, identified in the literature, will be used: a birth weight greater than 3500 g [14], weight gain in the first year of life (crossing of at least one percentile line of weight-for-length or zBMI percentile) [15], maternal or paternal overweight and obesity (BMI ≥ 25) [16], gestational smoking [17], and a low median household self-reported income [18]. Availability of at least one parent (caregiver) to participate in the study will be required.

#### Exclusion criteria

Young children with Prader-Willi syndrome or severe developmental delays or with obesity or severe obesity will be excluded, as they may require a different intervention; families who are not able to participate in English will also be excluded, as the intervention is only available in English; children with a sibling already enrolled in the study will be excluded to control for confounding variables; and families who reside beyond the Toronto Public Health catchment areas will be excluded from this study, as the intervention is delivered by Toronto Public Health nurses.

### Measurements

Eligible participants will be invited to complete questionnaires, physical measurements, accelerometry, and laboratory testing at baseline prior to randomization and at follow-up at 12 months. To comply with public health precautionary measures during the COVID-19 pandemic, if children and parents are unable to attend the primary care setting at enrollment, parents will perform height and weight measurements using standardized equipment with instructions sent to their home. Questionnaires and physical measurements will also be collected at 6 months. Questionnaires include measures of child’s mental health (Strengths and Difficulties Questionnaire), child’s development (Ages and Stages Questionnaire), child’s temperament (Child’s Behaviour Questionnaire), child’s dietary intake (NutriSTEP®), physical activity, sedentary behavior, sleep duration (Nutrition and Health Questionnaire), direct measure of physical activity, family psychosocial health (Depression, Anxiety, and Parenting Stress), parenting skills (Parenting Scale), and healthcare utilization. Physical measurements include anthropometric measurements to calculate zBMI and systolic and diastolic blood pressure. Blood samples for cholesterol, insulin, and glucose will be collected at baseline and the 12-month follow-up visit. Implementation feasibility and acceptability will be assessed by interviewing parents. Fidelity to the intervention will be assessed using fidelity checklists for all intervention core components, and cost will be collected from study investigators, participants, and administrative datasets.

### Clinical outcome variables

The primary outcome measure will be the difference in age- and sex-standardized BMI *z*-score at 12 months post-randomization in the intervention and control groups. zBMI will also be measured at 6 months post-randomization. BMI is the key obesity-related outcome used in prevention and treatment trials in obesity and is the Canadian standard for growth monitoring, using the World Health Organization Growth Chart [19, 20]. Secondary outcomes will include physical activity, screen time, sleep duration, eating behaviors, family psychosocial health, cardiometabolic risk (blood pressure, waist circumference, non-HDL, HDL, triglycerides, total cholesterol, glucose, insulin), and mental health outcomes in the children. Sociodemographic measures will be collected via a questionnaire. We will examine healthcare utilization outcomes for children and their mothers through secondary data in partnership with The Institute of Clinical Evaluative Sciences (https://www.ices.on.ca/). For a more detailed overview of all measures, please see Table 1.

**Table 1:** Outcome measures for the PARENT trial

Primary outcome		
Outcome		Outcome measure
Age- and sex-standardized BMI (6- and 12-month follow-up)		zBMI [32]
Secondary outcomes		
Child outcomes		Outcome measure
Child anthropometry and adiposity (baseline and 6- and 12-month follow-up)	Height Weight Head circumference Arm circumference Waist circumference	Measurement of child height (or length for children under 2) and weight is performed using standardized anthropometric protocols [33–35]. The WHO Growth Standards will be used to transform BMI to age- and sex-standardized *z*-scores [36]
Child cardiometabolic risk (baseline and 6- and 12-month follow-up)	Systolic and diastolic blood pressure	National High Blood Pressure Education Program (NHBPEP) [37]
	Non-fasting blood samples (only baseline and 12-month follow-up)	A trained health professional experienced with pediatric blood collection will collect 9–12 ml of blood. Topical anesthetic cream (e.g., EMLA or Ametop) will be offered to minimize discomfort from venipuncture. The blood samples include complete blood count, alkaline phosphatase, glucose, insulin, triglycerides, HDL, LDL, total cholesterol, non-HDL, ferritin, 25-OH vitamin D, CRP, and ALT [37]
	Diet intake, physical activity, sedentary behavior, and sleep duration	The NHQ is an age-specific TARGetKids! instrument based on the Canadian Community Health Survey and Canadian Health Measures Survey. Domains include outdoor free play [38]; sedentary behaviors, child daily screen time [39], and sleep quality and duration; and sociodemographic factors (parent self-reported income, maternal education, ethnicity, immigration status, and marital status). Parental health, mental health, exposures during pregnancy (e.g., nutrition, medication, and vitamin use), and family medical history
	Dietary intake	NutriSTEP® questionnaire for toddlers (18–35 months) and preschoolers (3–5 years) [40]. The NutriSTEP® is a parent-administered, validated 17-item nutrition screening tool that has been developed to assess nutritional risk and includes topics of dietary intake (including fruit and vegetable intake, fast food intake), physical activity and screen time, physical growth and development/weight concerns, and factors affecting food intake (food security, psychosocial feeding environment) validated to identify children with nutritional risk in multicultural Canadian children [40]
	Direct physical activity levels (only baseline and 12-month follow-up)	The Actical accelerometer device worn for 24 h on three consecutive days will provide direct measures of physical activity [41–43]
Child Mental Health and Development (baseline and 6- and 12-month follow-up)	Mental health	The Strengths and Difficulties Questionnaire (SDQ) consists of 25 items subdivided into four difficulties scales, emotional symptoms, conduct problems, inattention-hyperactivity, and peer problems, and a separate fifth strength scale, prosocial behavior [44]. The SDQ Total Difficulties Score (TDS) can be calculated as the sum of the scores for the emotional symptoms, conduct problems, hyperactivity in attention, and peer problem subscales (range 0–40). Scores from the SDQ have been found to be highly correlated with the Child Behavior Checklist which correlates with mental health disorders in adolescence and adulthood [45]. There are two SDQ versions, one for children ages 2–4 and one for children ages 4–17
	Development	The Ages and Stages Questionnaire (ASQ) is a screening questionnaire completed by parents, which identifies toddlers and preschool-aged children, at risk of a developmental delay in five developmental domains: communication, gross motor, fine motor, problem solving, and personal social behavior. Each domain consists of six questions about important age-specific developmental milestones [46]. The ASQ was developed and validated in the USA and is used worldwide. Psychometric characteristics of the ASQ were found to be excellent, with a sensitivity and specificity of 80% and 84%, respectively
	Temperament	The CBQ, a caregiver report measure, has been developed with the goal of providing a highly differentiated assessment of temperament in young children [47]. The Very Short Form of the CBQ (36 items) provides a comprehensive assessment of reactive and self-regulative temperamental behavior patterns in young children and is designed to assess 3 domains: surgency (12 items), negative affect (12 items), and effortful control (12 items)
Parental outcomes		Outcome measure
Parental anthropometry (baseline and 6- and 12-month follow-up)	Height, weight, waist circumference, BMI	Parent height, weight, and waist circumference will also be measured using standardized anthropometric protocols [33–35]. Reported weight and height of the other parent will be recorded if the second parent is not present
Parent health behaviors (baseline and 6- and 12-month follow-up)	Physical activity, sedentary time, and sleep duration	Parent-reported measures of physical activity, sedentary time, and sleep duration will be collected based on the Canadian Community Health Survey [38]
Parenting (baseline and 6- and 12-month follow-up)	Parenting skills	The 30-item parent-completed Parenting Scale is designed to help clinicians identify the discipline practices of parents of young children [48]. The scale measures three main parenting domains: laxness, over-reactivity, and verbosity [48]. A higher score indicates the “ineffective” end of the item
Family psychosocial health (baseline and 6- and 12-month follow-up)	Mental health	**Depression, Anxiety, Stress Scale (DASS-21)** The *DASS-21* is a 21-item parent-completed form designed to measure the negative emotional states of depression, anxiety, and stress. Each of the three DASS scales contains 7 items per scale [49] **Parenting Stress Index (PSI)** The 36-item, parent-completed PSI is designed to identify potentially dysfunctional parent–child systems and includes three scales: parental distress, difficult child characteristics, and dysfunctional parent–child interaction [50] **Patient Health Questionnaire (PHQ-9)** The Patient Health Questionnaire (PHQ-9) [51] is a self-administered multipurpose instrument for monitoring and measuring the severity of depression. The inventory scores each of the nine DSM-V criteria for depression as “0” (not at all) to “3” (nearly every day), and scores of 5, 10, 15, and 20 represent mild, moderate, moderately severe, and severe depression
Healthcare utilization	Total and mental health-related ED visits and hospitalizations for children and mothers	The study data will be linked to ICES, to gain access to information such as CIHI databases, specifically the Discharge Abstract Database (DAD) for hospitalizations and the National Ambulatory Care Reporting System (NACRS) for emergency visits for the purpose of identifying early life exposures in this cohort
Feasibility and acceptability		Semi-structured interviews with parents to assess their experiences of the overall conduct of the trial, perceived quality of the intervention, and barriers and facilitators to participation
Fidelity to the intervention		Fidelity checklists of core components for each group session and coaching visits will be completed by the public health nurses
Economic evaluation (12-month follow-up)		Cost-effectiveness will be expressed as the incremental cost-effectiveness ratio (ICER), which will be calculated by dividing the incremental costs between the treatment and control arms by the incremental change in the child’s zBMI between baseline and the end of the follow-up period

#### Implementation effectiveness outcomes

The feasibility, acceptability, and fidelity outcomes and their corresponding measures for the study are based on a working taxonomy of implementation outcomes [21]. Implementation fidelity will be assessed to capture adherence to the program core components, delivered as intended. A random sample of parents will be interviewed by phone regarding their experience (of the program and implementation).

#### Health service outcomes

A CEA will be used to determine the incremental costs of the parenting intervention compared to usual care in improving child’s BMI. Using patient-level data, the CEA will take a health system perspective and a 12-month time horizon. All costs and outcomes will be assigned to the family as the unit of analysis. Direct healthcare costs including costs of creating and executing the intervention and health service use during the study period will be collected from study investigators and participants. Individual health service utilization will be linked to administrative datasets for those healthcare visits which are reimbursed by the publicly funded system. Direct patient costs will include out-of-pocket expenses and childcare cost incurred from participating in the intervention. The parenting intervention will be micro-costed and will include all labor and supplies related to creating the program as well as ongoing delivery of the program. Intervention costs will be collected during the study interval and will be allocated across all participants.

### Study procedures

#### Eligibility and recruitment

Eligible participants will be recruited through the TARGetKids! practice sites in the Greater Toronto Area, Ontario, Canada, via research assistants and recruitment flyers and videos. To confirm and encourage parents and their children’s attendance at well-child check-up appointments, a research assistant will send one email reminder to the parent about their upcoming appointment 3–4 weeks before the scheduled appointment date. One week before their scheduled appointment, a research assistant will contact the parent via telephone, one time, to remind them of their scheduled appointment. Participants will be asked to complete an informed consent by an on-site research assistant if they choose to participate in the study. They will then complete baseline assessments and will be randomly assigned to the intervention or control group. All parents and children will have follow-up visits at 6 months and 12 months post-intervention by a trained research personnel, and all outcome data will be collected by trained TARGetKids! research staff embedded at each practice site.

#### Randomization and blinding

Individual children will be randomized based on a stratified randomization technique, ensuring equal recruitment of intervention and control participants for each practice site, executed via computer-generated random allocation sequence, in variable block sizes (i.e., 2, 4, 6). Contamination will be addressed by delivering the parenting group sessions after hours or online, and we will record all participants’ participation in other community-based services. To reduce the risk of bias, research assistants who will not be part of the delivery of the intervention will collect all outcome data, and data analysts will be blinded to group allocation. Parents cannot be blinded to the study allocation; however, they will be blinded that the primary outcome is zBMI.

#### Intervention

**Fig. 1:**
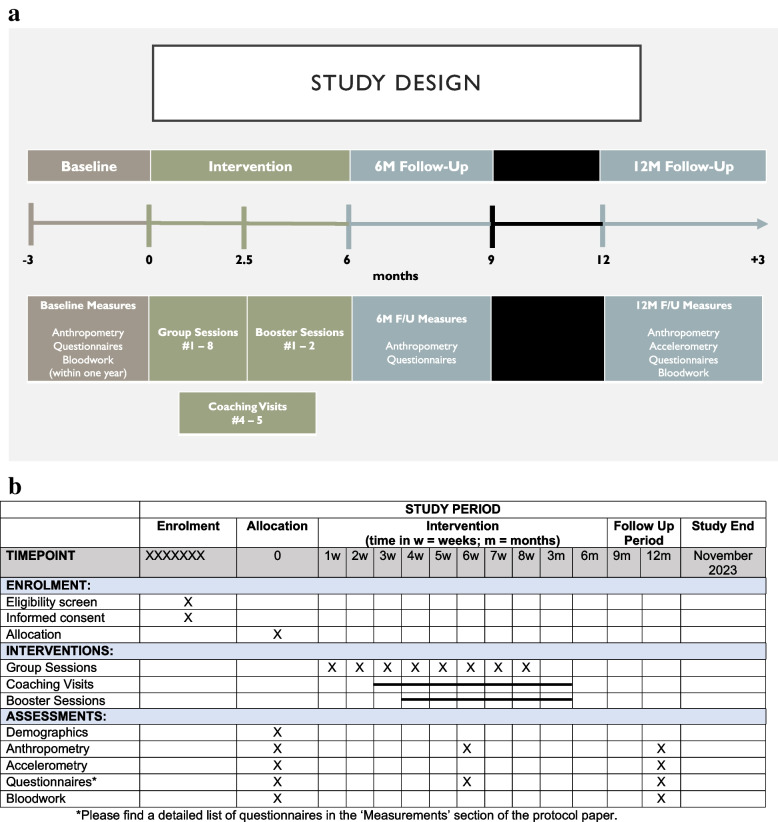
**a** Intervention and outcome measure timeline. **b** Overview of the study period (SPIRIT Fig. 2013)

The intervention is composed of eight 2-h weekly group-based sessions and 4–5 coaching visits over a 6-month period. To consolidate learning, 1–2 group booster sessions (1 h each) will be scheduled following completion of the eight parenting group sessions. The intervention curriculum was developed by a multidisciplinary team of experts in child growth and development, health and mental health, parenting, nutrition, and movement behaviors including physical activity, screen time, and sleep. For an overview of the study timeline, please see Fig. 1a and b.

All intervention components are facilitated by a trained public health nurse. Public health nurses [3] were selected for training and delivery of the intervention by the local public health unit, in consultation with the primary investigator, through an existing collaboration with SickKids Hospital and Toronto Public Health. The nurses were trained in parenting skills, and health behaviors, and had expertise in child obesity prevention and management. For this study, the nurses meet biweekly with the study team to discuss any feedback regarding the intervention. Parenting group session topics will include optimizing healthy behaviors and providing parents with parenting skills training. The parenting skills sessions are based on the Chicago Parent Program, an evidence-based program for parents and children to prevent and treat regulatory problems in young children and promote social, emotional, and academic competence [22]. General parenting topics to be covered will include persistence and emotion coaching, praise and social coaching, routines and rules, limit setting and follow through, and calm-down strategies. Due to the COVID-19 pandemic, all sessions will be delivered virtually over secure video conferencing (i.e., Zoom or Webex).

The coaching visits will be approximately 30–40 min long and delivered by a public health nurse. The coaching visits will integrate health messages and parenting skills addressed in other intervention components and encourage families to incorporate healthy nutrition and health behaviors individualized for their unique home and family environments through effective parenting practices learned in the program. The public health nurse will also link participants to local community resources where appropriate to support individual child and parental health and mental health identified during the coaching visits; referral will be facilitated by tools such as the Toronto Early Childhood and Family Resource System [23]. Finally, the coaching visits will be used to discuss participant progress, set and monitor personalized goals, and answer any questions. The criteria for a participant to discontinue the intervention are completed through participant request. The outcome data to be collected for participants who discontinue from intervention protocols is the same outcome data as those who attend the intervention parenting sessions (regardless of the degree of participation).

#### Control

The control group children and parents will receive the usual care. In Ontario, the usual care for this population consists of individual well-child health clinic visits guided by the Rourke Baby Record. Participants in the control group are being offered to participate in future virtual group sessions after the 12-month follow-ups are complete.

#### Data collection and management

Trained research assistants will facilitate the collection of questionnaire data, physical measurements, accelerometry data, and blood tests. These questionnaires will be completed on paper or electronically, according to parent preference. REDCap, a secure web-based application, will be used to capture data. Non-fasting blood work will be obtained by trained personnel according to the TARGetKids! protocol [24, 25]. To address public health measures during the COVID-19 pandemic, if children and parents are unable to attend the primary care setting at enrollment, parents will perform height and weight measurements using standardized equipment with instructions sent to their home. Blood tests are only collected if the child attends the clinic visit. A trained health professional experienced with pediatric blood collection will collect the small blood sample (approximately 9–12 ml/2–3 teaspoons of blood) from the child’s arm using a needle. Blood, saliva, and stool samples will be sent to the Mount Sinai Services laboratory where they will be examined. Test results will be kept in the database at the Applied Health Research Centre and a copy of the blood test results will be sent to your child’s doctor. Once these tests have been completed, any leftover samples will be transported to the Lunenfeld-Tanenbaum Research Institute for biobanking and future research. Future research may include hereditary genetic testing to look at whether a certain condition runs in families. The collection of these samples is a necessary part of this Registry. Samples will not be sold and will be used only for purposes approved by the Research Ethics Board. To protect the identity (privacy) of participants, samples will be de-identified and labeled with a study identification number, first name, birth month/year, and the date that the sample was collected prior to storage. This means that any identifying information, such as a participant’s first name and birth month/year, will be removed once they are received by the research team. Samples will be stored only with the participant ID. The researchers will collect information about participants’ medical history from their doctor’s records and will enter this information into an electronic database. The data will be securely stored and will be maintained by the Applied Health Research Centre at St. Michael’s Hospital. The database can only be accessed by people who are involved in this research. To gather information about healthcare use, research data will be securely transferred and linked to other databases, like the Ontario health administrative data held at ICES. Participant research data, which will be transferred, will include participant’s OHIP number, mother’s name, father’s name, child’s name, date of birth, and postal code. The information will be securely transferred as coded information, only for the purpose of linkage. Any direct identifiers such as the health card number or name will be removed or replaced with a code that is not known to study investigators.

#### Data monitoring

This intervention is considered to be of the low-risk nature. Based upon the Research Ethics Office at St. Michael’s Hospital (Toronto, ON) guidelines, because of this low-risk nature of the intervention, a Data Monitoring Committee (DMC) was not required.

#### Potential study harms

Evidence suggests that serious adverse events (SAEs) are not anticipated in this specific trial design. However, there is the chance that minor SAEs may occur. Minor SAEs include, but are not limited to, parent’s concern regarding confidentiality, parents questioning the reasoning behind their child’s recruitment for the study (i.e., Child is at risk for obesity), and any conflict that may arise in the group facilitation sessions. These potential minor SAEs will be reported to the Research Ethics Office at St. Michael’s Hospital.

#### Trial auditing

To review trial conduct, the PARENT Trial project management team meets on a biweekly basis where status updates are shared, and decisions are finalized around plans for the study. In addition, the PARENT Trial project coordinator meets with the TARGetKids! Operations Team on a weekly basis to share updates around the PARENT trial, discuss strategies and issues in relation to the cohort, innovate (with the other programmers, developers, coordinators, and clinical research specialists in attendance), and resolve any runway issues pertaining to the project database and/or data collection.

### Statistical analysis of clinical data

Appropriate descriptive statistics for baseline characteristics (frequencies and proportions for discrete variables; means and standard deviations for symmetric variables; and medians and inter-quartile ranges for skewed data) will be used to compare groups. The principle of intention-to-treat will be applied to the analysis of outcomes. Outcome variables will be compared between arms using ANCOVA with the corresponding baseline measure as the covariate. For the primary outcome, the following additional covariates will be included: age and each of the risk indicators—(i) birth weight greater than 3500 g, (ii) weight gain in the first year of life, (iii) maternal or paternal obesity, (iv) smoking during pregnancy, and (v) mean family income less than $80,000. Mean differences and corresponding confidence intervals will be calculated. A two-sided level of 0.05 will be applied for the primary outcome. To determine if the treatment effect on the primary outcome depends on baseline BMI, the interaction term between the treatment group and baseline BMI will be added to the covariate model. If this term is significant at the 0.1 level, then sub-groups determined by baseline BMI will be examined.

### Sample size

The sample size calculation is based on an ANCOVA for the primary outcome zBMI at 12 months, using the baseline zBMI as a covariate. The total sample size of 108 (54 per arm) is based on the following criteria: a two-sided, level 0.05 test of the null hypothesis of no difference; power of 0.80; smallest clinically important difference is 0.5 zBMI units at 12 months; between-subject standard deviation is 1 unit; and within-subject correlation is 0.638, derived from TARGetKids! dataset of subjects meeting criteria (*n* > 700). Lost-to-follow-up is estimated at 10% [26, 27]. Obesity intervention trials in young children with 15–50 children per group have demonstrated significant treatment effect sizes of 0.3–0.5 units of zBMI [10].

#### Feasibility and acceptability

Semi-structured interviews will be conducted with parent participants in the intervention and control groups to assess their experiences with the intervention, perceived quality of the intervention, and barriers and facilitators to their participation. Interviews will be recorded and transcribed, and thematic analysis will be conducted.

Fidelity to the core components of the intervention will be measured by the public health nurses following each group session and coaching visit using fidelity checklists and reported using descriptive statistics.

#### Economic evaluation analysis

Cost-effectiveness will be expressed as the incremental cost-effectiveness ratio (ICER) calculated by dividing the incremental costs between the intervention and usual care arms by the incremental change in the child’s zBMI between baseline and the end of the follow-up period. A probabilistic analysis using Monte Carlo simulation will be used to establish a point estimate and 95% confidence interval of the ICER. Extensive sensitivity analysis will be used to examine the robustness of the results and evaluate uncertainty in each of the parameters in the analysis. Ranges for the sensitivity analysis will be obtained from 95% confidence intervals generated from study data for each of the parameters. This analysis will be conducted in accordance with guidelines set out by the Canadian Agency for Drugs and Technologies in Health [28].

### Parent-oriented research approach

Parents participating in the TARGetKids! network have reviewed and provided feedback on the proposed study protocol and recruitment methods via a Parents’ Panel [29]. The Parents’ Panel was developed to engage parents involved in TARGetKids! research and gather their perspectives on proposed ongoing research. The Parent Panel will be engaged throughout the trial to support the interpretation of results and knowledge translation.

### Knowledge translation

TARGetKids! is a collaboration between child health researchers, primary care practitioners (pediatricians and family physicians), parents, and their children. This study incorporates an integrated knowledge translation approach through continuous engagement with practicing primary care clinics and public health nurses in the TARGetKids! network and with parents. At end-of-grant, study findings will be communicated to key knowledge users including parents, health professionals and organizations (primary care physicians and public health practitioners), decision- and policy-makers, and other researchers. The research team will actively engage with policy-makers early in the execution of the trial to ensure the program is scalable and feasible from a health policy perspective. Reporting of outcomes in peer-reviewed manuscripts will be guided by STaRI standards [30].

## Discussion

There are a few practical and operational issues that have been taken into consideration throughout the conduction of this trial. There have been challenges with coordinating the group sessions, to find a time that is appropriate for all parents attending these sessions. Due to COVID-19, the group sessions transitioned to online delivery which may have allowed an increase in program attendance. Recruitment into the trial was delayed due to COVID-19, as the primary method for recruitment is conducted in clinical practice. Participant uptake of coaching calls has also posed a challenge, as some participants are not always interested in attending these calls. To mitigate this, we have not made the coaching calls mandatory but encouraged by the public health nurse. One last challenge in terms of recruitment for this trial is the eligibility criteria. Participants included in this trial are those that have children who are at risk of developing obesity, which may present as a sensitive topic to discuss with a participant (parent) during initial enrolment. To aid in this discussion, we have implemented a script to guide our research assistants when communicating with the parent surrounding the topic of weight bias.

## Trial status

Protocol version number and date: 05 Jan 2021

Start date of recruitment: May 1, 2018

Estimated completion date of recruitment: April 15, 2023

## Data Availability

The final trial dataset will be available to study investigators and the Research Ethic Boards at all participating sites. The datasets analyzed during the current study and statistical code are available from the corresponding author on reasonable request, as is the full protocol.

## Abbreviations

ANCOVA: Analysis of Covariance
ICER: Incremental Cost-Effectiveness Ratio
RCT: Randomized Control Trial
TARGetKids!: The Applied Research Group for Kids
zBMI: Body Mass Index *z*-score
